# Essays on Life, Sleep, Pain, Etc.

**Published:** 1852-06

**Authors:** 


### REVIEWS.

Art. V.—*Essays on Life, Sleep, Pain, etc.* By Samul H. Dickson, M. D., Professor of Institutes and Practice of Medicine in the Medical College of the State of South Carolina, etc. Philadelphia: Blanchard & Lea. 12mo. pp. 301. 1852.

This is a pleasing little volume, and can not fail to be extensively read. The accomplished author has handled his subjects in a manner to recommend his Essays to the general as well as the professional reader; and they who dissent from his views on certain controverted points, or question the soundness of some of his speculations, must yet be interested in his facts and pleased by his polished, graceful style. While reading them we were often carried back in memory to the delightful hours which we spent, while still a boy, reading some of the "*Inquiries*" and the "*Lectures*" of our great Rush. There is in them the same freedom from the technicalities of science, the same lucid, simple style, the same appositeness of anecdote and illustration that we so much admired in the writings of that favorite author. They have the soul of science without its rigid form. The author places his subjects in a point of view to interest all readers.

The first essay treats of Life. In all the range of physiology or metaphysics, there are few more difficult subjects; and we are constrained to say that, while Dr. Dickson has exposed some of the errors of others who have written on the subject, the nature of the vital principle remains as obscure as ever. He is not more fortunate than the many other ingenious philosophers who have attempted to lift the veil that covers it. Life has been referred by some of the most eminent physiologists of the day to "organization." This view our author opposes, and in our judgment successfully. Life can not be the *result* of organization, for then organization could never occur. Life is the *cause* of organization; its special shape and form are the creation of the vital principle. There is a force—a *vis vitæ*—in the egg or seed, which determines and evolves all the parts of the organism. Organization is its conseqence. The germ is placed under favorable circumstances as to heat, moisture, and nutrition, and the pre-ordained animal or plant is developed in all its organs and textures. From generation to generation, this vital force continues to perpetuate the shape, the size, and the character of the parents in their offspring. The germs are alike as to size and structure--we can detect no difference between them-- and yet one grows into a fish, and another into a reptile— one into a bird, another into a quadruped. It is the principle of vitality, communicated by the parent to the germ, which determines the family or species. It is a *peculiar* principle. It has many attributes in common with electricity, but we can not agree with Humboldt, that it is but "a modification of the ordinary forces of matter acting under peculiar conditions." On the contrary, we would say with our author,—

It is not a *quality*, like hardness, softness, &c., but a power belonging to them derivatively, as I shall show hereafter, a property with which they are endowed on coming into existence in the forms known as organic. It is not oxygen, as Girtanner suggests; Matteucci gives abundant reasons for inferring that it is not electricity, as Abernethy and Wilson Philip were disposed to believe, and as it is now quite fashionable to suggest in the use of the terms electro-biology, &c.; nor is it a presiding genius, an archæus, an almost or quite intelligent agent, as Stahl and Prout have hinted; nor a mere pre-established harmony, as Aristoxenus and Leibnitz imagined; nor the product of organization, as Lawrence, Prichard, Mayo, and so many others maintain; nor is it to be found, as Cuvier, Bichat, and Coleridge intimate, rather darkly, I think, in the *tout ensemble* of the functions or anything else—to borrow the parliamentary expression of Mr. Joseph Hume, of financial fame, "the sum total of the whole."

If, as we have said, the vital force agrees in many things with chemical attraction and electricity, in many things it is opposed to them. It is superior to them, and controls them, and, in organized matter, they only become triumphant when it has become extinct. It has always appeared to us in the highest degree illogical to confound forces, the phenomena of which are so dissimilar. There is correlation between them, but they can not be the same.

After all, we have to go back to a great first cause for the source of this strange principle, which, out of bodies apparently identical, developes organisms so unlike each other. With Dr. Dickson, we "find a Supreme Being absolutely necessary in philosophy, as Robespierre did in social life." We refer the beginning and the transmission of this principle to Him. He created it, and by a power received from Him it has descended through countless generations, causing the species to perpetuate themselves each after their kind.

The very simplest of its manifestations are inexpressibly difficult to account for or comprehend; its very earliest influences altogether inscrutable; its apparent spontaniety of action, and its passive resistance to the effect of external agencies, equally inexplicable; and, as we contemplate it more and more closely, we are filled with a deepening conviction that there is nothing in the vast storehouse of nature more calculated to awaken intense curiosity, to invite assiduous investigation, and to give rise to solemn consideration, than the construction and movements of a living body, "fearfully and wonderfully made," and still more fearfully and wonderfully endowed with almost infinite capacities for action, for enjoyment, and for suffering. Let us humbly acknowledge that of this principle in the abstract we have hitherto formed very inadequate and unsatisfactory conceptions, and shall perhaps remain unsuccessful in our researches concerning it. It may be that He alone who possesses within himself this mysterious attribute, and who, of his infinite power and benevolence, has communicated it to a part of his creation, can fully comprehend its nature and its essence.

Truly, this power of life is a standing enigma. As beautifully expressed by Prof. Anstead, "it is no less a mystery to us now, than it was when man first speculated on its nature. We know not what it is, why it is, or how it is; we only know that it exists, and is everywhere present. At all degrees of temperature, from the surface of snow to boiling water of hot springs; in all soils, from the rich land of the tropics to the barren desert; on the whitened surface of pure salt, and on the naked rock on the mountain summit; in all degrees of light, from the full glare of sunshine to the darkest recesses of the rocky cavern; in air and in water; upon the earth and even beneath the earth; at all times, from the first introduction of living beings until now—we find pressing in on every side abundant evidence of this marvelous fact, this perpetual miracle. We are surrounded by the results of life; but we can not even imagine the nature of the broad line of demarcation, which seems, as it were, to form an impassable gulf between that which we call living, and that which is only dead matter." But if we may not attain to a knowledge of the abstract vital principle, there is no reason why we should not speculate about its manifestations. The subject is full of interest, and we will pursue some of the topics suggested by our author.

Dr. Dickson refers to the difficulty of drawing a clear line of demarcation between animals and vegetables, and shows that it is hardly less difficult to distinguish between instinct and the intellectual faculties. He even allows that bees, birds, and our domestic animals act from the promptings of hope and the suggestions of foresight. His language is:

We can know very little of the extension of feeling and sentiment through the lower orders of animals, for want of communication and intelligible expression; but the doctrine which ascribes to man exclusively the feeling of hope or anticipation must be abandoned when we reflect that all domesticated creatures expect their feeding time with habitual impatience, and press homeward before it arrives; not to dwell upon the promptings of what is called instinct, leading to the building of nests, the migrations of numerous tribes, the hoarding of food by several, and, among bees, the evidently purposed conversion of the immature insect of the hive into a great and worshipped queen, by a peculiar system of appropriate nutrition.

Our view of these phenomena is different. In the case of these animals, we conjecture, it is not an emotion allied to hope, or an exercise of mind like prescience, which leads to the actions described. They are very curious; they look very much like the operations of mind—so much that Todd and Bowman refer them to the mind of the Creator operating through these creatures. What could be more like the movements of an intelligent being than the wasp's burying in the sand with its egg the fly upon which the young maggot when hatched is to feed? For it is to be considered that the wasp itself feeds not upon flies or other animal food, but upon saccharine substances. Still we regard all these actions as purely *instinctive;* and suppose that the bee is no more obeying reason when it stores its cells, or feeds the embryo queen with food suitable for it, than the alligator is acting in obedience to rage when he turns his headless trunk suddenly in the direction of the hand that pricks it. They are actions which another part of the cerebral mass than that concerned in intellection, controls. They go on, just as respiration goes on, independently and in the absence of the hemispheres—under the influence of nervous centres which have no connection with mind or emotion.

On another point we are are compelled to dissent from the views of our author, who holds that there are some exceptions to the rule, as expressed by Richerand, that "plants may be considered the laboratories in which nature prepares aliments for animals." The earth-worm, and numerous other allied tribes, he says, "subsist upon materials derived from the mineral kingdom." "Nay," he adds upon the authority of Humboldt, "some of the wretched hordes of Southern America support their miserable lives, at least for a considerable portion of the year, upon a diet of clay." Nevertheless, we apprehend, the rule of Richerand will be found to be universal, and the exceptions merely apparent. The earth-worm finds organic matter in the mineral substances upon which it seems to subsist, and the clay is swallowed by the miserable people to give distension to the stomach. It has no power of supporting life, and the savages would die as soon with it as without it, if they derived nourishment from no other source. Many of the lowest tribes of animals feed upon organic matter in a state of decomposition, as it is just about to return to the inorganic kingdom, and thus restore it, by a shorter route, to the living world; but none, it is believed, find nutriment in substances strictly mineral. Plants obtain their aliment from inorganic matters, and out of them elaborate the food of animals. We take it, few points in physiology are better settled than this. We believe the rule is without an exception. The mushroom grows luxuriantly near decaying organic matter; but it is the products of the decomposition—the carbonic acid, ammonia, etc.—and not the organic matter itself that it subsists upon.

Dr. Dickson is no believer in spontaneous generation; but, at the same time, he does not accept the principle, *omne ab ovo.* Life, he agrees with Cuvier, "can be born only of life;" but then, in his view, it does not "necessarily originate through the medium of a seed, or an egg, or germ of any kind." He remarks:

The decay of organized bodies seems, as one of its uniform results, to effect or offer the separation and elimination of organized corpuscles. The vitality which has been diffused through the whole mass, and does not desert it at once or simultaneously in all its parts, may *in transitu* fix in some of the myriads of disintegrated particles, effecting the diversified organization which we see, and producing the infinite variety of infusory animalculæ and vegetable minims, algæ or tremellæ, the absolute nature of which, as I have already said, is undetermined Nay, it is perhaps in itself indeterminate, for the primary globule or minute cell seems, whether vegetable or animal, to be identical in structure and composition, as far as these are or can be known.

The meaning of this passage, if we understand the learned author is, that a large animal suffering putrefaction is broken up into a great number of small ones! In another place he says, "I see no reason for limiting the transmission of life through identical or even similar forms." An organism of great size, he supposes, may eliminate "organized corpuscles," that grow into animals wholly unlike the body that gave them birth; not only unlike, but vastly inferior. "The derived life," as he expresses it, "originating in the processes of decay and decomposition, is always inferior, base, and fragmentary; worthy of its source in the compost heap and charnel-house; sinking and retrograding, as it would seem, of necessity; never tending upwards, or self-elevating."

We should rather say, that these "organized corpuscles," which putrefying bodies afford, are ova deposited in them by insects. We confess, the idea that such corpuscles may be "eliminated" by decaying animal matter strikes us as most irrational. All analogy condemns it. It is better than the Lamarckian hypothesis, in that the corpuscles are not held to be developed into higher organisms. If we had to choose between the two doctrines, we would rather believe in the downward tendency of such generations, than be obliged to accept the speculation, that they pass gradually from this low state into nobler forms, finally becoming men; but either view is bad enough. Both doctrines rest upon a slender basis, which the current of scientific discovery is continually wearing away. One strong hold after another has been given up by the advocates of spontaneous generation, until now they have hardly any ground left to stand upon. What was obscure has been rendered clear; what seemed inexplicable has been explained by the persevering researches of naturalists. Botanists and physiologists have followed up the process of generation, and have succeeded in showing, that in almost all the cases where plants or animals have appeared to be generated spontaneously, they came in reality from progenitors. The ova or seed that produced them have been detected in nearly all these instances, and there is no room to question that those in which they have not been found are an exception to the law. If the fungi and algæ, the rotifera and entozoa can be shown to perpetuate their species by germs, there is no reason to doubt that all the allied, and much more all the higher tribes, are propagated after the same fashion. In a word, we must accept as a fundamental truth of physiological science, "that *every living organism has had its origin in a pre-existing organism.*" There is no longer any excuse for rejecting or doubting it. What Carpenter expresses in reference to it is coming to be the creed of the whole scientific world. That able writer says: "The doctrine of 'spontaneous generation,' or the supposed origination of organized structures *de novo* out of assemblages of inorganic particles, although at different times sustained with a considerable show of argument, based on a specious array of facts, can not now be said to have any claim whatever to be received as even a possible hppothesis; all the facts on which it claimed to rest having either been themselves disproved, or having been found satisfactorily explicable on the general principle *omne vivum ex ovo.*"

Our author's hypothesis is but a modification of this doctrine, and therefore obnoxious to all the objections urged against it. The difference to us seems trifling between the origin of life from inorganic matter, and its generation among particles of putrefying animal bodies.

The subject of the second essay is *Sleep*, the repose of the nervous system. Where there is no nervous system there is no sleep. The "sleep of plants" is like their "love," a thing that has no existence except in the imagination of poets. It is an animal function, and those organs only that minister to animal life are subject to it. The heart and the lungs never sleep.

It is still an undetermined question in physiology what causes sleep, which Carpenter defines to be "the state of suspension of the sensory and motor functions." What is the condition of the brain in sleep? Is the state the same in all kinds of sleep, natural, morbid, and mesmeric? We give our author's remarks on this subject:

In examining closely the circumstances presented during the state of sleep, we shall discover, I think, that the concurrence of two obvious elements is necessary to its production. Any living fibre, when subjected to inspection, nay, any living globule of any living fluid, presents, when in action, the condition of vital tension as the most evident proof of its activity; an erethism, a constitutional or compositional excitement, greater or less in proportion to the degree of its vitality and the energy of its action. But the tension of the tissues, of which the globules of living fluids are destined to form parts by deposition and fixation, can not be always sustained at an equable rate, but must undergo alternately at proper periods a comparative relaxation. Throughout the waking hours, various portions of the nervous centres are in varying states of tension and erethism, and a certain degree of relief is obtained from time to time by each, through the removal or change of impression and action from one portion to another. After a while, however, they all grow weary, and, as the old writers correctly imagined, undergo a collapse, by the subsidence of the tension or erethism of which I have spoken. But the cerebral organ is contained in an unyielding case of bone, and can not thus shrink, unless the space to be made vacant by its shrinking be filled up in some mode. Hence arises the intermixture of the second element whose concurrence I contemplate as necssary, the determination of blood, namely, to the cerebral mass, and its congestion in the larger vessels of the brain, chiefly as I suppose the veins and sinuses, whose structure and arrangement are somewhat peculiar, and well adapted to receive and sustain this congestion. It is true that, between the immediately investing membranes of the brain, and its unyielding bony case, there is interposed for purposes of support and defence the cerebro-spinal fluid, which varies in amount according to a thousand varying contingencies of the animal economy. But the rapid, nay, the instantaneous transitions from the sleeping to the waking state, do not admit of any satisfactory reference to such changes in quantity, which must require notable and comparatively prolonged intervals of time, occupied by secretion and absorption.

The fact of cerebral collapse during sleep is established by observation in cases where, the bone being deficient, the organ was liable to external pressure. Blumenbach states that he himself witnessed in one person a sinking of the brain whenever be was asleep, and a swelling when he awoke. Dendy relates the story of a woman who, at Montpellier, in 1841, had lost part of the skull, the brain and its membranes being laid bare. When she was in deep sleep, the brain lay in the skull almost motionless; when she was dreaming, it became elevated; and when her dreams were on vivid or animating subjects, as proved by her afterwards relating them, but more especially when she was awake, the brain was protruded through the cranial aperture. In another case, Combe observed, through an opening in the skull, that the brain was elevated during an apparent dream.

He goes on to speak thus of sleep induced by artificial means:

This theory or hypothesis of the physical causation of natural sleep applies well to the explanation of some of the enforced or artificial sleeps. Suppose either of these two elements producible at will, and it tends to generate the other; the undue predominance of either constituting the special character of the sleep. Thus, if you diminish the cerebral tension, no matter how, by a long story, a dull sermon, or (*e. g.*) a tedious essay; or by fatigue, which exhausts the nervous power generally; or by wasting disease ("I must sleep now," exclaimed the dying Byron); or by inanition, or large drafts from the fluids of the body in any mode—congestion takes place in the cerebral veins and sinuses, the eye reddens and is suffused, the sight grows dim, the face, except in the bloodless, flushes somewhat, the lids droop, the muscles relax, and slumber invades us.

If, on the other hand, you promote in any way the entrance of blood into the vessels of the head, while you avoid any excitement which may arouse the mind, and thus oppose a cerebral tension to the dilatation of the vessels within the cranium, you will bring on sleep. Thus act rocking in a cradle; the oscillations of a hammock; the gyrations of a circular swing; almost infalliby the motion communicated by a body moving round upon its own centre, as when lying with head to the circumference, and feet to the centre of a platform, revolving like a millstone. Mesmeric sleep has thus been accounted for. A position or attitude of muscular relaxation is arranged; all mental action repressed—all sensation, and every impression, interrupted or veiled; a quiet attention directed to the conjuror, who makes no sound, and avoids all rude movement; under these circumstances, the cerebral erethism of thought, motion, aud emotion gradually subsides, while the vascular determination is in a gentle way aided by the fixation of the eye, on which so much stress is laid by Baird, of Newcastle, and Ratcliffe Hall. As to the effect of ether, chloroform, and other anæsthetics, from which exulting humanity has already derived so much benefit, and indulges so much larger and hopeful anticipations, it presents a very complicated and difficult problem. Perhaps it is, in part, owing to the quick absorption of the exhaled fluids by the blood in the lungs, which is transmitted to the delicate and yielding texture of the cerebral fibres, impregnated with their highly elastic vapor, adapted to exert within the vessels a powerful, though very transient intravascular pressure. They are all, I believe, of this rapidly vaporizable and elastic character, undergoing great expansion by moderate increase of temperature.

Certain pathological facts are consistent with, if they are not rather confirmatory of, this view of the condition of the brain in sleep. If Solly be right, and there is no positive reason to pronounce him otherwise, delirium tremens, of all maladies known to us the one in which sleeplessness is most specially the prominent element, presents a remarkably anemic state of the brain; the deficiency of blood in the organ totally prohibiting the vascular congestion which I maintain to be a necessary requisite in sleep; there being, at the same time, a most extrordinary intensity of erethism or cerebral irritation. Chloroform, by subduing the latter, removes the former evil, and has been found among the most certain and prompt remedies for this wretched effect of intemperance.

Marshall Hall contends that convulsion can not happen until congestion in the head has been produced by the spastic contraction of the muscles of the neck, the platysma myoides particularly, which prevents the return of blood downwards through the large veins. Now sleep, or a condition strongly resembling it, either immediately precedes or attends simultaneously, or follows an attack of convulsions. M. Hall advises that the epileptic be not suffered, for this reason, to sleep soundly. We infer that a patient sleeps through a convulsive paroxysm too, because he is happily insensible at the time or retains no memory of consciousness; and all are familiar with the profound slumber which usually terminates these dreaded invasions, of whatever character, hysteric or epileptic.

The relations of Sleep to disease are curious. Thus, some maladies select the night season as the period of attack, and others seem to prefer the day. Asthma, for example, "almost always assails during the first heavy sleep on lying down." The invasions of cholera are most frequent at night, and at a late hour, after sleep has lasted a good while. "Colic awakes the patient often. Diarrhea and dysentery begin when the night's sleep is about to end." Some fevers are most apt to invade in the day time; "yellow fever often arouses the startled slumberer." "In thirty years of inquiry," says Dr. D., "I have heard of very few instances, not more than *six* in all, of the supervention of *intermittent*, the occurrence of a *chill*, in the state of sleep. Malarious *remittent* seldom or never, I know no example, attacks during sleep."

Sleep has hygienic bearings of an important character. Few things exert a more controlling influence upon digestion than sleep, and many cases of dyspepsia may be traced to its indiscreet abridgment. Its effect upon the state of the mind is not less decided. In a state of sleeplessness, "the temper becomes harsh and sour; the man is petulant, testy, unreasonable, and liable to great depression of spirits." Let him enjoy a night of sound repose, and he will wake up an altered being. His irascibility will be gone, and his spirits have become buoyant. Many persons are thus vibrating between these extremes of comfort and wretchedness, because they do not properly heed the admonitions of "nature's sweet restorer." Every one is aware how salutary is this repose of the nervous system to the sick. No one will question that there is great truth in the remarks of our author, that—

Sleep, quiet and profound, is, in a very large proportion of diseases, not only a good symptom, but positively and beyond question remedial. It suspends many irritative, and some inflammatory affections. *Catarrhal* annoyances yield, for the time, to its gentle sway; it is the only hope of relief in numerous forms of agonizing *cephalalgia*. *Neuralgic* anguish of every kind is not only suspended while it continues, but, when regularly recurrent, has its tenacious periodicity interrupted most efficiently. Indeed, in almost all periodical affections, some advantage is sure to be derived from the use of narcotics, so timed as to put the patient soundly to sleep at, or a little before, the hour of their return. This is remarkably notable in the instance of malarious intermittents, which I have many scores of times put off by a soporific dose of opium. Indeed, I think most of the therapeutic benefits of opium, ascribed to its diaphoretic property, flow from its delightful influence in procuring sleep, a state almost incompatible with certain modes of irritation and inflammation; and this leads me to hope much similar advantage from the enlarging list of anæsthetics, in all forms of disease of which the combination of these elements constitutes the prominent feature of the first or invading stage.

One of the most dreaded of human maladies is unquestionably often a consequence of morbid vigilance. Dr. Brigham, who investigated the causes of insanity with remarkable assiduity and success, sets this down as paramount among them. According to his observation, sleeplessness continued beyond a certain period is pretty certain of ending in insanity.

In perfect sleep, there can be no consciousness, no dreaming, and from that state, in which the whole cerebral mass appears to be at rest, up to the state of somnambulism, there are endless grades of mental activity. The only difference between dreaming and somnambulism is, that the individual is more awake in the latter condition. Answers may be obtained, and action aroused by whispering in the ear, "low enough not to awaken, but loud enough to affect the sense of the sleeper." Beattie, who is quoted by our author, relates an instance of an officer played upon by his comrades, who induced him, by whispers, to go through the whole ceremony of a duel, till waked by his pistol.

'He was told that he had fallen overboard, and began to imitate the movements of swimming: being warned that a shark was following him, he dived from his couch upon the floor. When he was notified that a battle had commenced, and was raging around him, he ran away."

A capital subject this officer would have made for the "*electro biologists*." His gifts were native to him and needed not the "passes" of mountebanks to awaken them. If it were more generally known that this state is one easily excited in many individuals by natural processes, the feats of these charlatans would soon cease to be the wonder of gaping multitudes. Dr. Dickson relates some remarkable examples of somnambulism, from which we select the following :—

*The Lausanne Transactions* contain the case of a somnambulist who sometimes opened his eyes to examine where he was, or where his inkstand stood, and then shut them again, dipping his pen every now and then, and writing on, but never opening his eyes while he wrote on from line to line regularly, though he corrected some errors of his pen and in spelling; "so much easier was it for him to refer to his *ideas* of the positions of things," says the relator, "than to his *perceptions* of them." Darwin mentions a young lady who occasionally in her sleep sung and recited poetry. In repeating some lines from Pope, she had forgotten a word, and began again, endeavoring to recollect it. It was shouted aloud several times in her ear, but to no purpose; yet, after many trials, she at length regained it herself.

From Hone's *Every Day Book* I take the following strange case, related since by Dendy: "A lad, Davis, æt. sixteen years, rose, got his whip, put on a spur, and went to the stable; not finding his saddle, he inquired for it; being asked what he wanted with it, he replied—'to go his round;' being a butcher's boy. He then got on the horse without a saddle, and was going out, but was stopped and taken off by force. Mr. Ridge, a surgeon being sent for, stood by him some time, during which he thought himself at the turnpike gate, and took sixpence out of his pocket to pay the toll. It was returned to him, but he insisted on its being changed, and resisted an effort, made in jest to cheat him. While Mr. Ridge was bleeding him, he joined in the conversation. After an hour he awoke, went again to sleep, and was well next morning."

It is a question in which most persons have felt an interest at one time or another, how much ought a healthy man to sleep? We quote from Dr. D. in answer to the question:

The average proportion of time thus employed by our race may be stated pretty fairly, I think, at *one third.* The allotment of Sir William Jones, slightly altered from an old English poet, does not depart much from this standard:—

> "Seven hours to books, to soothing slumber seven;
> Ten to the world allot, and all to heaven."

The busy engagements of ambition and avarice may induce men to substract more or less from their due repose, but any considerable deduction must be made at great risk to both mind and body. Sir John Sinclair, who slept eight hours himself, says that, in his researches into the subject of longevity, he found long life under every circumstance, every course of habit; some old men being abstinent, some intemperate; some active, and some indolent; but all had slept well and long. Yet he gives a letter from a correspondent, recording the case of an old man of ninety-one who had slept through life but four hours a day. Alfred the Great slept eight hours, Jeremy Taylor but three. Dr Gooch tells us of an individual who slept only fifteen minutes in the day; but this is scarcely credible. Bonaparte, during the greater part of his active life, was content with four or five hours' sleep; the same is said of Frederick the Great, and of John Hunter. I know familiarly a person whose average has been even lower than this; I have heard his wife say that they were married four years before she had seen him asleep. Seneca is quoted as telling the incredible story of Mecænas, that he had passed three years without sleeping a single hour. Boerhaave says of himself that he was six weeks without sleep, from intense and continued study. Statements like these demand close examination and clear proof.

The propensity to sleep when it comes on in a natural way is one of the hardest to resist, and when brought on by *cold* is irresistible. "He who sleeps," said Solander in his famous antarctic expidition, "will wake no more," and yet a few hours after was so overcome by the desire that he begged his companions to indulge him just a little while. The certainty of dying from the indulgence was not enough to keep the philosopher awake. How overpowering the disposition often is when circustances are unfavorable, most churches on any fair sabbath afternoon might testify; but there are few who do not know from experience how hard many times the balmy influence is to be won when most importunately wooed. In that queer book, so rich in thought and beautiful expression, the *Doctor*, Southey has given the various expedients for inducing sleep, and we quote the passage, with which Dr. Dickson's second essay concludes:

I put my arms out of bed; I turned the pillow for the sake of applying a cold surface to my cheek. I stretched my feet into the cold corner. I listened to the river, and to the ticking of my watch. I thought of all sleepy sounds, and all soporific things; the flow of water, the humming of bees, the motion of a boat, the waving of a field of corn, the nodding of a mandarin's head on the chimney-piece, a horse in a mill, the opera, Mr. Humdrum's conversation, Mr. Proser's poems, Mr. Laxative's speeches, Mr. Lengthy's sermons. I tried the device of my own childhood, and fancied that the bed revolved with me round and round. At length Morpheus reminded me of Dr. Torpedo's Divinity Lectures; where the voice, the manner, the matter, even the very atmosphere, and the streamy candlelight, were all alike somnific; where he, who by strong effort lifted up his head, and forced open the reluctant eyes, never failed to see all around him asleep. Lettuces, cowslip wine, poppy syrup, mandragora, hop pillows, spider's web pills, and the whole tribe of narcotics up to bang and the black drop, would have failed, but this was irresistible; and thus, twenty years after date, I found benefit from having attended the course.

*Pain* is the subject of the third essay, the object, origin, and end of which is one of the dark problems about which metaphysicians have speculated in vain. Physicians have ever held it to be a part of their function to annul or alleviate it. How strange that anesthetics should have been objected to by some on the ground, that they are in contravention of a law of nature making pain a necessary evil! Dr. Dickson has not much patience with those who contend that pain is a useful guide to the surgeon in his operations. He says—

I should be very unwilling to employ for my own person any one who needed such warning or guidance as might be derived from my agonies while under the edge of a knife, or bound and dragged by his mechanical apparatus. His knowledge of physiology and anatomy, if as thorough as it ought to be, will require no such aid.

Dr. Dickson quotes a case of natural anesthesia reported by Prof. P. F. Eve. The man was operated upon for cataract; he was immoveable, and said he felt no pain. The remainder of the history is as follows:

Having had a finger injured in a rencontre, he bit it off himself, and spat it on the ground, not liking its appearance. He had an ulcer on the toe and foot for three years; from first to last, he said it never gave him the least pain. He had an abscess in his hand, which threatened his life, involving the whole forearm and arm with enormous swelling; it was opened repeatedly and freely, but during the whole time he experienced no pain. His neck having been once pustulated by tartar emetic, he did not feel it at all, but ordered the application to be renewed. Prof. E. made three incisions on the back of his neck to relieve erysipelatous inflammation. "He was so unconscious of the operation, that, after it was performed, he asked that it should be done, that he might turn over in bed. He told his physician that he had never suffered pain from any cause whatever, until his last illness. He was a man of great probity, and never boasted of being insensible to pain. He had been at one time addicted to the free use of alcoholic potations."

The subject of the next essay is *Intellection*. No subject is more interesting, and it is here treated with great ability. Mind is referred by all to the brain, which is coming to be regarded by anatomists as a great ganglion or collection of ganglions, duplex in its formation, and susceptible, like other double organs, of disease in one hemisphere while the other is in a healthy condition. Upon this rests the doctrine of phrenology, "so clearly founded in well-ascertained truth, but pressed by its advocates into so many extremes of error." Dr. D. regards as questionable the occurrence of an absolute *monomania*, remarking—

I have never certainly seen any instance in which a *single faculty* or power of the mind was perverted *exclusively;* that is, allowing all others their normal range and capacity. I have examined many such, and have always found some collateral disorder and confusion. In medical jurisprudence, it is extremely unsafe to suggest or maintain the views which are becoming so prevalent in the present day on this point. Unless there were certain other exhibitions of mental aberration, I can not agree to hold guiltless a thief, simply because he exhibits an inordinate thieving propensity—nor a murderer, because he is urged on by a homicidal inclination—any more than I would acquit of the guilt of assault a morbidly pugnacious man. The doctrine is untenable and dangerous, and will, if pressed, lead to a cruel and savage reaction, as in the case of Baker of Kentucky—where a furious maniac suffered the penalties of the law while howling defiance to all laws; a scene of inhumanity sufficient to have "hung the heavens with black."

Yet, believing in the duality of the brain he sees no reason to doubt that insanity may depend upon a diseased condition altogether confined to one hemisphere.

In such cases, alternate thoughts and actions will exhibit the contrasted character and state of the mind, and at times present a varying preponderance of power in the sound over the diseased hemisphere, and *vice versa*. Now, in these examples of double existence, we shall aid the sane man in his endeavor to govern the insane portion of himself, by retaining over him the influence of motive, in the accustomed and efficient forms of "hope of reward and fear of punishment."

He remarks thus on the propensity to suicide—

The mildest and most inoffensive person—one of the most regular in his habits, the most exact in his perceptions, the most upright in business, the most capable of transacting his affairs, and correctly dividing his property after death—may labor under an irresistible propensity to suicide. Have we forgotten the English minister, under whose government England attained her loftiest pinnacle of power, the termination of whose ambitious and highly successful career is told in a single epithet, alliterally compounded by one of his bitterest political enemies, "carotid artery cutting Castlereagh!" A second may be the subject of a vehement propensity to injure and destroy others, and yet shall be acute in business, and clear in his natural, moral, and religious views; and a third shall be under the absolute dominion of fancy, and subject to the strangest hallucinations, while capable of the full performance of all the offices of his station—a penetrating reasoner, a sound adviser, a valuable friend.

We confess we would rather adopt the prevailing belief, that the propensity to self-destruction is an evidence of insanity. The author continues—

Phrenologists have lately been engaged in hunting for an organ of self-destruction, and they will probably find it, as they have found so many other organs which must be separate, as they infer, because of the distinctive character of the faculties and propensities which depend upon their activity. But Phrenology, though she has long located confidently the organs of destructiveness and combativeness, has not saved a single life by raising the friendly warning of danger from heads of evil conformation. She is only "wise after the event." So we must not indulge in too sanguine expectations of aid from her, in anticipating the inclination to shorten life, but proceed to examine whether there are any other indications by which we may be guided to our task of prevention. Suicide, the most desperate of human acts, is often the result of motives apparently trifling, and in such cases is apt to be committed with great apparent deliberation. The slighter shades of ennui have occasioned it, as in Dr. Darwin's patient, who complained to him that "a ride out in the morning, and a warm parlor, and a pack of cards in the evening, comprised all that life affords." We hardly wonder that, after fifty years of such a life, he got tired of it and shot himself. Montaigne and others afford us cases of suicide from the most transient slights and mortifications. These excite our surprise as well as horror; but there are many others which move all our sympathies. When we hear of the volutary death of a woman who has lost her honor; of a monarch dethroned; of a warrior beaten in his last battle, as when Brutus falls upon his sword after the fatal field of Philippi; of a merchant irretrievably ruined in fortune and credit; of a physician whose professional reputation is hopelessly blasted, as in the melancholy case of the attendant on the Princess Charlotte, we are ready to acknowledge that, however we may be shocked at the deed, it is suggested by feelings common to our whole race. "Time, the great consoler," can alone blunt the acuteness of mental sufferings in instances like these, and restore the tolerance of life. It is the *judgment* which is unsound in the suicide. Death is chosen as a refuge, because of the assumed impossibility of enduring the train of evils in prospect; just as the duellist goes out to meet his antagonist, because, if he refuse, he will be made to groan under an insupportable burden of obloquy and disgrace. A singular instance of suicide from disordered *perceptions*, however, came under my own notice a short time since, attended with such remarkable confusion and even uncertainty of personal identity that it deserves to be recorded. I was called hastily to see a man who had cut his own throat with a razor. It was exceedingly difficult to dress the wound of the patient on account of his earnest outcries against the ruffian who had held him down upon the floor and murdered him.

Whenever a sufficient motive, then, presents itself to prompt to suicide any unhappy sufferer from whatever cause, he should be carefully watched, and, if need be, restrained by efficient control. Beyond this, there are other circumstances that ought to excite suspicion. Burrows says that the propensity is most strongly marked in the eye and countenance, and that the look can scarcely be mistaken. I wish he had described it, so as to enable us to recognize it, which I confess I have failed to do. It has occurred to me to lose two patients in this way, whom I was attending for ordinary chronic complaints, without remarking in their appearance or manner anything striking or unaccustomed.

But we can extend our remarks upon this admirable work no further. The essays on *Hygiene* and *Death* are quite equal to any others in the volume, and if we had the space we would be glad to make numerous quotations from them. As it is, we can only commend them with the rest to the careful perusal of our brethren, assuring them that they will find the series one of the most interesting that the medical press of our country has lately brought forth.

